# Use of Human In Vitro Gut Specimens for Translational Neurogastroenterology and Motility in the 21st Century

**DOI:** 10.1111/nmo.15022

**Published:** 2025-04-28

**Authors:** Dmitrii Pavlov, Fievos L. Christofi

**Affiliations:** ^1^ Department of Anesthesiology, Wexner Medical Center The Ohio State University Columbus Ohio USA

**Keywords:** enteric neuropathy, human ENS, human intestinal specimens, intestinal surgical specimen, neurogastroenterology and motility

## Abstract

There is a huge gap in our understanding of the human ENS and translating data from mice to humans that is important when developing targeted therapeutics. The ENS or “human little brain in the gut” is easily accessible for study in GI surgical or biopsy samples. This mini review is focused on the use of human gut specimens in translating laboratory data on ENS and enteric neuropathies in neurogastroenterology and motility from mice to humans. Availability of viable human gut samples, in combination with technological advances in innovative recording techniques and new in vitro models provide powerful ways to study neural activity and secretomotor function or monitor motility in health and disease with exquisite sophistication and precision. Electrophysiological recordings, optical recordings with voltage‐sensitive dyes, or Ca^2+^ imaging (in adult or fetal gut) is used to study neural activity in human ENS in health and disease. ‘First in man patch clamp recordings’ is possible in isolated networks of human myenteric ganglia, opening the door for patch‐seq. The human ENS at single cell resolution (snRNA‐seq) revealed cell‐diversity, similarities and differences between human and mouse in vitro. Visceral afferent recordings are used for mechanosensation and pain signaling in humans. Stem cell therapies may hold future promise for patients with enteric neuropathies. A greater focus on the human ENS and enteric neuropathies (i.e. IBS, FD, postoperative ileus, CIPO, chronic constipation, Hirschsprung Disease, infection, gastroparesis, Parkinson's disease, IBD, visceral pain) is one important step for consideration in developing potential therapeutics before proceeding to more expensive and complex clinical trials in patients to treat GI Disorders and Diseases.

## Graphical abstract

In vitro studies with GI surgical specimens or biopsy are a critical step in translating preclinical studies in animals to humans before testing potential therapeutic interventions in expensive clinical trials. Routine availability of human gut specimens, in combination with technological advances and powerful new in vitro models of the human ENS from surgical tissue or biopsy specimens, is providing new insights and advances in the field of neurogastroenterology and motility for GI disorders and diseases such as IBS, FD, postoperative ileus, chronic constipation, CIPO, infections, gastroparesis, Parkinson's Disease, Hirschsprung Disease, IBD, and visceral pain. Guidelines outlining a comprehensive and standardized approach to the use of human samples in translational studies are urgently needed.

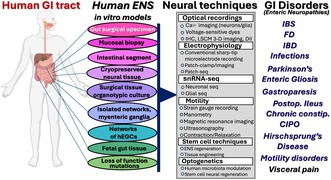

**Summary**

- There is a huge gap in our understanding of the human ENS and translating data on ENS from mice to humans that is important when developing targeted therapeutics.
- The use of human gut specimens is an important step in translating laboratory data on ENS and enteric neuropathies from mice to humans in neurogastroenterology and motility.
- Routine availability of human gut specimens, in combination with technological advances and powerful new in vitro models of the human ENS developed from surgical tissue or biopsy specimens, is providing new insights and advances in the field of neurogastroenterology and motility for GI disorders and diseases such as IBS, FD, postoperative ileus, chronic constipation, CIPO, infections, gastroparesis, Parkinson's Disease, Hirschsprung Disease, IBD, and visceral pain.

## 1 Introduction

The use of animal models to advance human medical studies dates back to 6th century B.C. Greece and has been pivotal in science for millennia [1]. Various advancements, from early surgical techniques to the discovery of insulin, are contributions of animal studies [2]. However, the modern translation of animal model research to human subjects has been unpredictable, and there is concern about the clinical validity, reproducibility, and application of animal research to evaluate the human condition. In this minireview, we aim to address the use of human gut specimens in translating data from animal studies to humans in the field of neurogastroenterology and motility. Ultimately, translating findings from mice to humans is a complex but critical aspect of biomedical research [3] that deserves more attention.

Translatability of laboratory data on ENS from mice to humans is important when developing targeted therapeutics [4]. Unlike human brain, the ENS or “human little brain in the gut” is easily accessible for study from GI surgical or biopsy samples. Recent efforts with pluripotent stem cells hold promise for innovative studies to better understand the development, function, and regeneration of the ENS and motor function [5, 6]. Enteric glia, immune cells, and neurons contribute to neuroinflammation, enteric neuropathy, and ENS dysfunction, causing dysmotility in animal models [4, 7, 8, 9]. To what extent this knowledge is translatable to man is largely not known [4, 6]. Translating in vitro findings to humans is challenging for many reasons. Species differences may exist in function, structure/anatomy, receptors, immune responses, distinct pathophysiological mechanisms, signaling pathways, and diseases that need to be explored further for meaningful interpretation of animal model studies [1, 3]. Simply, the human is not always a good model for the mouse [10, 11]. An article in nature reviews bioengineering [10] on “human disease models in drug development” points out that biomedical research is moving from animal models towards human disease models in drug development. Costs for drug development are escalating with an all‐time low success rate, and most drugs fail in clinical stages despite proven efficacy and safety in animal models. There are many potential reasons for this, but one reason is that proceeding to clinical trial relies almost exclusively on animal models that may potentially have low predictive value to humans. A comprehensive review on this topic points out that “biomedical research is undergoing a paradigm shift towards approaches centered on human disease models owing to the notoriously high failure rates of the current drug development process” [10]. Interspecies differences between animals and humans are discussed in the next section.

Our focus in this review is to discuss how in vitro human gut models of the human ENS are being used to further advance our knowledge of the ENS in normal and diseased conditions, identify species differences and similarities, and evaluate the translatability of animal studies to humans. There is a critical gap in our understanding of the human ENS in health and disease. Enteric neuropathy is emerging as an important pathogenic mechanism in GI diseases and disorders. In the last 25 years, in vitro models of the human ENS and innovative techniques and approaches have been developed to study the human ENS in health and disease [7, 12, 13, 14]. Advances in technology have made it possible to study the human ENS with a similar level of sophistication as in animal studies, to carry out mechanistic studies in healthy and diseased gut to address important questions in neurogastroenterology. In this minireview, we will highlight key examples and applications of human gut samples to illustrate the progress made over the past 25 years in translating findings from animals to humans. Important differences between mouse and human guts comprise the chemical coding of neurons [10, 11, 15], the ratio of glia: neurons per ganglion (i.e., 7:1 ratio) [16, 17, 18, 19], their clustering based on their RNA fingerprint [17, 20], the rate of metabolic activities [21], the composition of microbiota [22] and immune system [7, 8, 23].

Thus, it is no wonder that there is growing concern that because of the significant differences that exist between the mouse and human gut, mouse models of gut disorders may not always adequately recapitulate the human pathophysiology. Studies that pioneer molecular and functional characterization of human enteric ganglia have started to appear [9, 18, 24, 25, 26]. Moreover, a wide range of invasive studies cannot be performed in humans in vivo, highlighting the necessity of human ENS cell cultures or human gut tissue research and further development and validation of preclinical animal models [10, 14, 27]. Several papers provided proof for both variations and conserved gene expression patterns in enteric glia and neurons between mice and humans [9, 11, 28, 29, 30, 31]. Our minireview will stress the use of human gut samples to study the human GI tract in health and disease. It is emerging as a critical step in translating findings from animals to humans before eventually testing a therapeutic intervention in clinical trials. More translational studies are desperately needed.

## 2 Inter‐Species Differences Between Animals and Humans

It is noted that major drivers for this transition are the limitations of animal models, which, despite remaining the gold standard in basic and preclinical research, suffer from interspecies differences and poor prediction of human physiological and pathological conditions. Differences in microbiome composition and its metabolites, like short‐chain fatty acids and other metabolites, have been identified between mouse, rat, non‐human primate, and human feces [32], differences in the physiology of gut motility [33], and notable differences in size and complexity of human ENS versus mouse, i.e., 168 million enteric neurons in humans vs. 2.5 million neurons in mouse [34]. While we agree that improving the design of animal studies could potentially increase their predictive value, we contend that even the most rigorously conducted preclinical studies in animals often fail to replicate the complexities of human physiology. For instance, species differences in gut motility, receptor distribution, and the gastrointestinal microbiome can significantly influence experimental outcomes [35, 36]. Moreover, even with high‐quality animal models, there are notable instances where treatments that show promise in animal studies fail in human clinical trials (e.g., the failure of NK1 receptor antagonists in clinical trials despite promising preclinical results in rodent models) [36].

We observed notable species differences in humans (versus mice) in P2X receptor subtypes in submucosal neurons [21]. Deiteren et al. [37] identified differences in the role of histamine receptors (H1–H4 in the GI tract and receptor distribution between mice and humans, including differences in the ENS expression and function of these receptors). In humans, ENS expression of H3 is lower than in mice, and their role in GI motility is less pronounced compared to mice. In fact, the ENS in mice tends to be more sensitive to histamine activation than in humans. In particular, H3 receptors exert a more pronounced influence on transmitter release and smooth muscle tone in mice compared to humans. This difference likely accounts for the more pronounced effects on histamine motility observed in rodent models. Moreover, histamine plays a modulatory role in humans, whereas in mice it may directly affect gut motor function and even contribute to pathophysiology in disease models like IBS or IBD. Other receptors differ as well between mice and humans, and species differences in purinergic receptors complicate the use of purinergic drugs for GI disorders or diseases. A comparative study of voltage‐sensitive dye responses in guinea pig and human ENS revealed that all four histamine receptors‐H1, H2, H3 and H4 are involved in excitatory responses in the human submucous plexus [38]. In contrast, guinea‐pig ENS neurons primarily respond to histamine through H2 receptors, with less involvement of H1, H3 and H4 receptors. This study has also shown that certain H1 receptor antagonists exhibit higher binding affinities in guinea pigs compared to humans, suggesting structural differences in receptor binding sites. In mice, the ratio of glia to neurons is reported to be 1:1, whereas in humans, the ratio is reported to be 7:1, with glial cells being more abundant than neurons of the ENS [29]. Shared and disparate subtypes of neurons were identified in mouse and human ENS using single‐cell transcriptional profiling [30].

## 3 Enteric Nerves, Secretion, Fluid Regulation and Diarrhea

Ove Lundgren [39] demonstrated that “ENS” is responsible for at least 60% of the gut secretory response. In vivo agents that can cause massive secretion include bacterial enterotoxins (such as cholera toxin, *Clostridioides difficile*, *Escherichia coli*), noxious agents (ethanol, Na deoxycholate, and chemotherapy), invasive organisms (*rotavirus, nippostrogylus brasilliensis, salmonella typhimuvirus RS*), inflammatory mediators, anaphylactic hypersensitivity, or postoperative ileus. Therefore, the ENS has implications for fluid regulation and diarrhea and is a potential site of action of drugs. Lundgren [40] further emphasized the crucial role of the ENS in mediating fluid and electrolyte secretion in the small intestine. The ENS is involved in a wide range of secretory diarrheal conditions, including *Vibrio parahaemolyticus infections*, IBD, and certain medications [39, 40]. The ENS mediates these responses through complex neural reflexes and signaling pathways that enhance fluid and electrolyte secretion in the gut. Neuroimmune interactions play a significant role in the pathophysiology of secretory conditions. By focusing on the ENS, new treatments could be designed to more effectively manage and treat different forms of diarrhea [41]. *Ca* ^*2+*^ *recordings in intact human submucous plexus*. Pioneering studies on the human ENS were done using Laser Confocal Calcium imaging in intact human submucous plexus preparations (hSMP) to provide an in vitro model to study ENS neural circuit activity in secretomotor reflexes. For example, fiber tract stimulation was used to study synaptic transmission and the neuropharmacology of purinergic receptors [21, 42] in intact submucous ganglia using a Laser confocal Ca^2+^ imaging technique to monitor neural activity. Our studies revealed adenosinergic inhibition of neurotransmission [42]; and the detailed neuropharmacology of purinergic receptors in human submucous plexus involved in secretomotor function, including P2X1, P2X2, P2X3 channels, P2Y, and A3 metabotropic receptors in neurotransmission within the human submucous plexus [21, 42]. Using live‐cell calcium imaging, it was demonstrated that activation of these receptors leads to significant Ca^2+^ transients in enteric neurons. Endogenous purines were found to be critical regulators of neurotransmission in the human ENS with notable species differences in P2X receptor subtypes [9, 43]. These channel receptors are sensitive to inflammation, and the in vitro model can be used to compare healthy and diseased tissues for purinergic signaling. The potential for developing purinergic drugs for GI diseases such as IBD has been reviewed [9]. In addition, simultaneous measurements of motility by sonomicrometry and secretory (Isc) responses [44] were done in animals—and could easily be adopted to humans. Our pilot/feasibility studies showed that coordination of motility and secretion could be studied by strain gauge recordings of serosal contractions and ISC secretion induced by mucosal stroking (*360° rotation*) in full‐thickness human gut specimens (Christofi, unpublished observations).

## 4 Advances in Neural Recordings of the Human ENS

Routine recordings with voltage‐sensitive dyes, Ca^2+^ imaging, or electrophysiology are used to monitor neural (or glial) behavior in normal or diseased human gut tissues (i.e., *intact micro‐dissected, myenteric, or submucous plexus preparations, isolated ganglia or cells*) and these studies have yielded important new insights on the human ENS. In vitro studies in human tissues yielded information on the function, dysfunction and effects of drugs on secretion, motility or enteric nerve activity.

### 4.1 Use of Voltage‐Sensitive Dyes for Human ENS Functional Studies [13]

Optical recording with voltage‐sensitive dyes by Schemann's group proved to be a reliable technique for capturing neuronal responses to stimuli with high temporal resolution. The use of voltage‐sensitive dyes provided several advantages, including the ability to monitor activity from multiple neurons simultaneously and to capture fast electrical events that might be missed with traditional electrophysiological techniques. This approach also allowed for non‐invasive recording, preserving the integrity of the tissue. Recordings from human myenteric neurons using voltage‐sensitive dyes have been instrumental in gaining important new insights into our understanding of the human ENS in IBS [12], autoimmune diseases [45], studies on obesity [46] and actions of natural plant extracts on human ENS function [13, 41] with therapeutic potential. The ENS is implicated in the pathogenesis of various autoimmune diseases [47]. Alterations in ENS function and structure have been observed in autoimmune conditions, suggesting that the ENS may contribute to the disease process. A review by Annaházi and Schemann [47] focused on the human ENS for autoimmune diseases and IBS. Buhner et al. [12] also carried out voltage dye recordings to show activation of human enteric neurons by supernatants of colonic biopsy specimens from patients with IBS. Differences in responses to supernatants from IBS‐D or IBS‐C colonic biopsy specimens, suggested subtype‐specific differences in the pathological mechanisms and the nature of soluble mediators involved. Soluble factors from the colonic mucosa of IBS patients can directly influence ENS function, potentially contributing to the symptoms of IBS, such as altered motility and pain perception. Another application is the study of immune regulation of the ENS [48]. Mast cells and neurons in the ENS closely interact, with mast cells influencing neuronal activity and vice versa. These interactions are crucial in regulating GI motility, secretion, and immune responses. Dysregulation of mast cell‐ENS interactions is implicated in various GI disorders, including IBS, inflammatory bowel IBD and functional dyspepsia. Mast cell activation can lead to the release of mediators such as histamine, tryptase, and cytokines, which affect neuronal signaling and contribute to symptoms like pain, altered motility, and inflammation. Targeting the mast cell‐ENS axis could offer new therapeutic approaches for managing GI disorders. Therapies aimed at stabilizing mast cells, blocking their mediators, or modulating neuronal responses to mast cell activity may potentially alleviate symptoms and improve patient outcomes.

Use of voltage‐sensitive dyes for human ENS functional studies offers advantages such as high temporal resolution, non‐invasive recording, and the ability to study human‐specific pathologies and immune interactions in a proof‐of‐concept approach. It provides more relevant insights into conditions like IBS and GI disorders that could provide a basis for further testing in both preclinical and clinical trials to develop novel therapeutics.

### 4.2 Conventional Intracellular Recordings From Myenteric Neurons

There is sparse data available on the electrophysiological and neuropharmacological properties of human enteric neurons. Simon Brookes and co‐workers have published several excellent papers using conventional intracellular recording techniques in GI surgical tissues in the study of myenteric neurons of the human ENS [14, 49, 50, 51]. These are complex and technically challenging recordings, but such elegant studies are providing important new information on the chemical coding, pharmacology, excitability, and synaptic transmission in the human ENS, in healthy and diseased/compromised surgical tissues (i.e., toxic response to chemotherapy). Notable differences exist in AH neurons between species. In this regard, although Dogiel type II neurons have been identified in both guinea pig and human ENS, the strong correlation between Dogiel type II morphology and AH electrophysiological properties observed in the guinea pig ileum does not seem to apply to human ENS [52].

This technique holds great promise in future studies on the translatability of findings from animals to humans and studies on disease states, although it remains a formidable challenge to record from many neurons to sufficiently power studies for a rigorous analysis of the population response, and progress has been hampered by this. Patients undergoing anticancer chemotherapy often experience gastrointestinal side effects, but the exact causes are unclear. It is uncertain whether this treatment directly affects the enteric nervous system [50]. Colon specimens from chemotherapy‐treated and non‐treated patients who underwent colorectal resections for carcinoma removal were compared. Intracellular recordings from myenteric neurons and immunohistochemistry were performed in whole mount preparations. Myenteric S neurons from chemotherapy‐treated patients were hyperexcitable with a lower rheobase and threshold to evoke action potentials compared to non‐treated patients. This study suggested both functional and structural changes in human myenteric neurons in colon specimens from patients undergoing anticancer chemotherapy. These changes may contribute to the gastrointestinal symptoms experienced by chemotherapy‐treated patients.

Using human tissue for electrophysiological studies of the ENS provides insights into the chemical coding, pharmacology, and excitability of human neurons, which may differ from animal models. It allows for the study of disease states, such as chemotherapy‐induced changes in myenteric neurons, providing more accurate information on human‐specific responses that may not be easily replicated in animals.

### 4.3 Patch Clamp Recordings From Isolated Networks of Human Myenteric Ganglia

The ‘first in man’ in vitro patch clamp recordings' were done in our laboratory from intact networks of human myenteric ganglia (nhMPG) isolated from surgical tissue (and is also possible from mucosal biopsy; DDW 2022—Basic Science Plenary session). The potential exists to adopt this technique for Patch‐seq as in brain slices and apply snRNA‐seq to identify subtypes of cells in isolated networks to complement Patch‐seq. For RNA‐seq analysis, patch‐clamp recordings were performed from brain slices using glass micropipettes filled with an intracellular solution containing RNA‐stabilizing agents. After electrophysiological characterization, RNA was aspirated by applying light suction until the cell had visibly shrunken and downstream transcriptomic analysis was carried out [53, 54].

In future studies with further refinement of our technique, it should be possible to carry out electrophysiological, transcriptomic, and morphological profiling of single neurons (or glia) using patch‐seq as was done in brain slices in animals [51, 53] that may be applied to healthy patients or patients with a GI disease. Using human tissue for patch‐clamp recordings from isolated myenteric ganglia allows for direct study of human‐specific neuronal properties and cell subtypes, which may not be accurately reflected in animal models. The combination of electrophysiological, transcriptomic, and morphological profiling offers deeper insights into both healthy and disease‐affected human ENS, facilitating more relevant and proof‐of‐concept research on GI disorders.

## 5 Human Enteric Glial Networks (hEGCs)

Enteric glia may represent a new Frontier in Neurogastroenterology [29] and Clinical Target for IBD [24]. Enteric glial cells (EGCs) play essential roles in modulating motility, maintaining gut homeostasis, and contributing to neuroinflammation in intestinal diseases and motility disorders. Damage to the intestine induces a reactive glial phenotype known as “enteric gliosis,” but the molecular identity of the inducing mechanism and triggers of “gliosis” are poorly understood. Use of isolated human enteric glial networks in culture (hEGCs) is suitable to study mechanosensation, Ca^2+^ waves, purinergic signaling, “reactive glia” in response to inflammation, GI diseases like IBD, or functional bowel disorders like postoperative ileus. A recent review [55] focused on the impact of inflammation on enteric glia reactivity in response to diverse insults such as intestinal surgery, infections (HIV‐Tat‐induced diarrhea, *C. difficile infection*, endotoxemia), GI diseases, and functional GI disorders [55]. Some of the findings in preclinical animal models may be translatable to humans, raising the possibility of testing therapeutic interventions in future clinical trials. The hEGCs model provided a translational link in those studies.

We are studying neural pathogenic mechanisms of postoperative ileus (POI) and dysmotility by combining studies in glial‐specific cKO mice for various targets and translational studies in human gut tissues. In vitro studies utilize hEGCs, intestinal organotypic cultures (IOC) of longitudinal muscle—myenteric plexus preparations (LMMP) tissues, and early/late samples of jejunum from human pancreatectomy to study the impact of surgical trauma and gut manipulation‐induced POI. Pathogenic targets of POI identified in ‘reactive glia’ in mice and humans include interleukin‐1 [56], P2X [7] and ET_B_ receptors [57], Connexin‐43 hemichannels (unpublished observations, Christofi), and mechanosensitive channels. For example, a recent study by Schneider et al. [7] found that ATP activation of a p38‐dependent MAPK pathway triggers cytokine release and a gliosis phenotype in murine and human EGCs. Receptor antagonism and genetic depletion studies revealed P2X2 as the relevant ATP receptor involved in enteric gliosis. Pharmacological screenings identified the drug Ambroxol as a novel P2X2 antagonist, which could prevent ATP‐induced enteric gliosis, inflammation, and protect against dysmotility. Ambroxol was also found to abrogate enteric gliosis in the human intestine in organotypic culture exposed to surgical trauma. This suggested that interventions targeting enteric glial P2X2 receptors during surgical trauma may represent a novel therapy for treating POI and immune‐driven intestinal motility disorders [7, 8, 24, 29, 54, 56, 58].

Gut surgical manipulation and excessive mechanical forces during the surgery is implicated in human POI. Low Pressure Pneumoperitoneum in laparoscopic surgery is the target of a clinical trial to protect against postoperative ileus (NCT05344417, Christofi PI). Pathogenic mechanisms are explored in human gut samples.

Our human ENS studies in isolated networks of myenteric ganglia or cells in collaboration with Dr. Shanthi Srinivasan on an NIH‐funded study are focused on ‘neuronal ferroptosis’ as a mechanism of iron overload, enteric neuropathy, and dysmotility in overweight and obesity. An abstract on this work combining animal and human studies is submitted to DDW2025.

Abdominal pain can result from altered neuro‐immune interactions in the gastrointestinal tract, but the specific signaling processes connecting immune activation with visceral hypersensitivity are not fully understood. The study by Grubišić et al. [54] hypothesized that enteric glial cells serve as a link between the neural and immune systems of the gut and that communication between enteric glia and immune cells influences the development of visceral hypersensitivity. Studies in hEGCs provided proof for the involvement of macrophage colony‐stimulating factor in the pathogenic mechanism in human Crohn's disease.

Application of human enteric glial networks (hEGCs) in culture is providing new insights into human‐specific mechanisms involved in neuromodulation of motility, neuroinflammation, and reactive enteric gliosis in GI diseases, which may not be fully represented in animal models. Studies in hEGCs allow for a better understanding of human conditions like postoperative ileus, inflammatory bowel disease, and visceral hypersensitivity that could potentially lead to the identification of more relevant therapeutic targets and interventions for human patients and also serve as a proof‐of‐concept approach.

## 6 In Vitro Human Motility Studies [1, 4, 14]

A recent review article by Wattchow et al. [14] nicely describes the importance of in vitro studies of human colonic motility. Early studies using organ bath techniques provided knowledge about the basic contractile properties of colonic muscle and the role of neurotransmitters and highlighted the importance of enteric neurons in regulating colonic motility. Such translational research has been critical in identifying key motility patterns and their physiological relevance in humans. The paper discusses the use of high‐resolution manometry and advanced imaging techniques (e.g., MRI, CT scans) to study colonic motility in vivo. These techniques have provided insights into the spatial and temporal patterns of colonic movements, such as peristalsis and segmentation, including the coordination of muscle contractions and the role of neural and hormonal control. It also discusses how abnormalities in these motility patterns are associated with disorders such as IBS and chronic constipation. Understanding of colonic motility has direct implications for diagnosing and treating gastrointestinal disorders. The review highlights the importance of tailored therapeutic approaches based on specific motility patterns and underlying pathophysiological mechanisms and calls for further research to integrate findings from different levels of study (molecular, cellular, tissue, and whole organ) to build a more cohesive understanding of colonic motility. It advocates for the development of new technologies and methods to bridge the gap between laboratory findings and clinical applications. In vitro motility studies in human surgical tissues [14, 16] is providing important insights into drug actions on neuromuscular function and gut reflexes. Spatiotemporal imaging has increased the level of sophistication for the analysis of intestinal movements, and further technical improvements are possible by combining this technique with impedance manometry, magnetic resonance imaging, electrophysiology, and ultrasonography. Stem cell‐based therapies and tissue‐engineering approaches with embryonic or human pluripotent stem cells may prove useful in the future for treating gut dysmotility disorders (i.e., Hirschsprung's disease or chronic intestinal pseudo‐obstruction [59]).

In vitro human motility studies provide critical insights into human‐specific colonic motility patterns, neurotransmitter roles, and neural control, which may differ from animal models. These studies, using advanced techniques like high‐resolution manometry and spatiotemporal imaging, help improve the understanding of GI disorders such as IBS and chronic constipation. It can also be considered a proof‐of‐concept approach.

## 7 First in Human Visceral Afferent Recordings From the Colon, Appendix and Rectum

Electrophysiological recordings of human colonic afferents in freshly isolated *human colon* or *vermiform appendix—mesentery tissue* provide useful models to study the properties of human visceral afferents [42, 60] and pain signaling in humans in vitro, before pursuing expensive clinical trials. This research represents a pioneering “first‐in‐man” investigation of the mechanosensitivity of colonic afferents in human subjects in response to controlled distensions and contractions of the colon. Human colonic afferents exhibit distinct responses to mechanical stimuli such as stretching and pressure. The characterization of colonic afferent mechanosensitivity has significant clinical implications. By understanding how these nerves respond to mechanical stimuli, researchers and clinicians can better comprehend the mechanisms underlying gastrointestinal pain and develop more effective treatments for conditions such as IBS or other functional bowel disorders.

Recent studies by Ng et al. [49] and Peiris et al. [61] confirmed, for the first time in humans, the anatomical and functional existence of extrinsic afferent nerves linking the rectum to the CNS. These nerves exhibited spontaneous multiunit afferent activity, which was recorded and analyzed, establishing their presence and functionality. The researchers characterized the chemo‐ and mechanosensitivity of these extrinsic rectal afferents. They found significant increases in peak firing rates following exposure to capsaicin and “inflammatory soup” and identified mechanosensitive “hot spots” in the rectal afferents. The thresholds for mechanosensitivity decreased after exposure to the ‘soup,’ indicating heightened sensitivity under inflammatory conditions. The study highlighted significant differences between rectal and colonic afferents. While spontaneous activity was recorded in most rectal nerves, it was rarely observed in colonic nerves. Additionally, the mechanosensitive thresholds in colonic nerves were much higher, suggesting distinct electrophysiological properties and sensitivities between rectal and colonic afferents.

**FIGURE 1:**
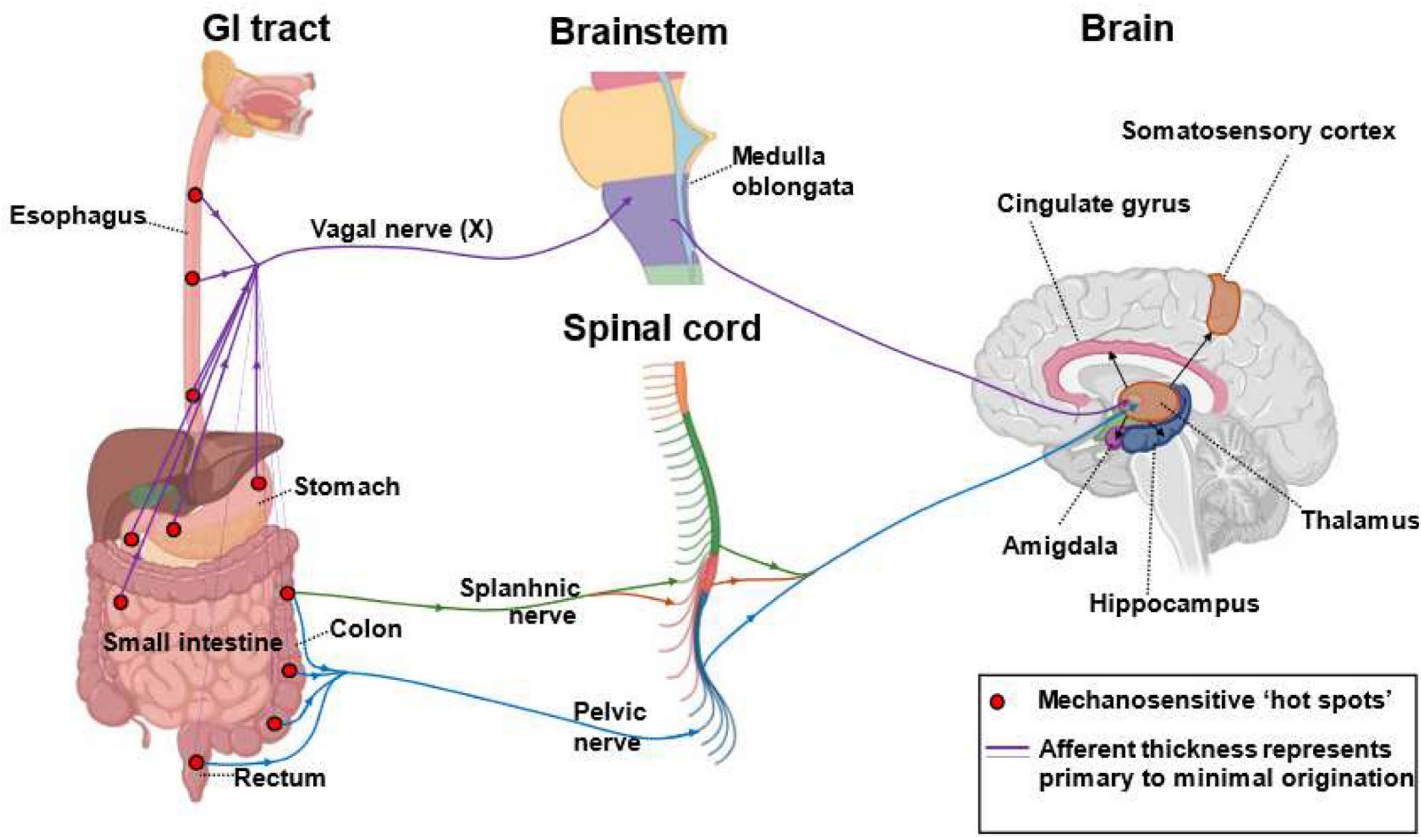
Schematic representation of mechanosensitive “hot spots” in the gastrointestinal tract and pathways of rectal and colonic extrinsic afferents to the central nervous system. The figure illustrates the key anatomical sites of mechanosensitivity along the colon and rectum, highlighting their functional relevance. Hot spots (i.e., receptive fields) for mechanical stimulation are depicted by red dots throughout the GI tract. Hot spots are innervated by the Vagus nerve or abdominal branches of the splanchnic and pelvic nerves. Vagal afferents project to key limbic structures via the brainstem, while abdominal nerves mainly travel via the spinal cord.

Figure 1 illustrates the locations of mechanosensitive “hot spots” in the gastrointestinal tract and the pathways of rectal and colonic extrinsic afferents to the central nervous system. Table 1 summarizes key electrophysiological properties of human visceral afferents, with notable differences between colonic and rectal afferent sensitivity. Less information is available for appendiceal afferent sensitivity.

**TABLE 1:** Key electrophysiological properties of human visceral afferents.

Stimulation parameters	Colonic afferents sensitivity	Rectal afferents sensitivity	Appendiceal afferents sensitivity	References
Mechanical stimulus (vfh) threshold	High (strong/painful distension)	Low/mild (distension and stretch)	No human data	[49, 61]
Mechanical stimulation	Slow‐adapting response, to sustained luminal stretch	Rapid and precise response to rectal filling	No human data	[49, 61]
Baseline firing rate	0.14 Hz [49], 2 Hz [61]	0.37 Hz	2 Hz	[49, 61]
Capsaicin sensitivity	Moderate (Peak, 9 Hz)	Low (Peak, 6 Hz)	High (60 Hz)	[49, 61]
Inflammatory soup	Low (Δν* = ↑2 Hz)	Low (Δ*ν* = ↑3 Hz)	Very high (Δ*ν* = ↑70 Hz)	[49, 61]

Abbreviations: Δν*, change in frequency (Hz); vfh, von Frey hair.

Differences between serosal afferent sensitivity and muscular afferent sensitivity in human visceral afferent recordings is summarized in Table 2 for spontaneous activity, mechanical (VFH), stretch, and algesic mediator responses (i.e., ATP, bradykinin, histamine, PGE2, 5‐HT, etc.). A more recent study identified distinct subpopulations of human visceral afferents comparable to those in animals. This includes a population of polymodal nociceptors responding to bradykinin, ATP, capsaicin, histamine, etc. Differences in sensitivity to visceral afferent stimulation are summarized in Table 2 [62]. Visceral afferent sensitivity to mechanical or chemical (i.e., algogenic) stimulation (i.e., von Frey hair) depends on sex differences, age differences, the specific region of the intestine being investigated, and whether it is normal or diseased (i.e., IBD) tissue. Human serosal visceral nociceptor mechanosensitivity is attenuated by TRPV_4_ antagonist (HC067047) or 5‐HT agonist (tecaserod) used in treating pain in IBS, highlighting the importance of this human in vitro model for the identification and evaluation of novel analgesics and further basic research in visceral pain [62]. Overall, data support differences between colonic and rectal afferents, although it should be acknowledged that the exact mechanistic pathways leading to these differences remain unclear and deserve further investigation. Clear differences in sensitivity were observed between the two types of visceral afferents [62].

**TABLE 2:** Visceral afferent recordings in human intestine [62].

Stimulus	Serosal afferent sensitivity	Muscular afferent sensitivity
Mechanical (VFH)	High	Low
Stretch	Low	High
Algesic mediators (ATP, bradykinin, histamine, PGE2, 5‐HT)	High (polymodal)	Low
Spontaneous activity	Low	High

Human tissue provides a more accurate model for studying visceral afferent responses and pain signaling in patients, as it reveals distinct mechanosensitivity and electrophysiological properties of human colonic and rectal afferents that may not be fully reflected in animal models. At its core, this is a proof‐of‐concept approach.

## 8 Use of Optogenetics in Animal Models to Study Important Human Gut Microbiome and Stem Cell Regeneration

The application of optogenetics provided valuable insights into gut physiology and neurogastroenterology research in animal models [63]. There are several studies applying human microbiota in optogenetic models or using human stem cells to regenerate the ENS in a Hirschsprung disease mouse model using optogenetics in the investigation. An optogenetics‐integrated organ culture system to explore mouse ENS in human‐like gut environments [64]. Cultured enteric cholinergic neurons that express light‐sensitive channelrhodopsin‐2 (ChR2) were illuminated with blue or yellow light, in parallel to luminal infusion of a human symbiont *Clostridium ramosum*. Combinatorial optogenetic stimulation and bacterial infusion remodeled neuroimmunological responses induced by optogenetic stimulation alone, mainly affecting genes and pathways related to cytokine production and cell‐to‐cell contact. Authors rightly point out that this approach may provide a suitable platform to test neuroimmune connections and modulation by drugs, microbes and their metabolites.

While the microbiome and metabolome offer exciting therapeutic potential, translating findings into clinical practice faces several challenges. Microbiome composition varies widely, influenced by genetics, diet, and environment, complicating the identification of universal interventions that can overcome interindividual variability. The application of the next generation of multi‐omics approaches (genomics, metabolomics, and proteomics) with advanced imaging and clinical longitudinal data will likely unravel the complex interplay within the gut‐brain axis [65].

A paper by Fattahi et al. [66] demonstrated that ENS progenitors derived from human pluripotent stem cells (hPSCs) could differentiate into functional enteric neurons in a Hirschsprung disease (HSCR) mouse model (Ednrb mice). Various neurotransmitter phenotypes, including 5HT^+^, GABA^+^ and NOS^+^ neurons, were identified in differentiated hPSCs. ChR2 was genetically expressed in hPSCs under the control of the human synapsin promoter. They probed the functionality of enteric neurons by assessing in vitro connectivity with smooth muscle cells (SMC, i.e., muscle contraction). Optogenetic stimulation induced contractions of co‐cultured SMC, which are closely associated with ChR2^+^neurons. Moreover, an in vivo engraftment and migration of hPSCs‐derived ENS precursors rescued disease‐related mortality in Ednrb mice. This human stem‐cell platform provides a novel approach for the study of human ENS development and presents cell‐ and drug‐based strategies for the treatment of HSCR.

## 9 The Human ENS at Single Cell Resolution With Single Cell Nuclear RNA‐Sequencing (snRNA‐Seq) Analysis

The application of single cell sequencing technology is advancing our understanding of human enteric neuronal and glial diversity for future studies in GI diseases [11, 19, 53]. Such studies identified > 20 subtypes of neurons and 3–7 types of enteric glia [11, 15, 17, 19, 20, 30]. Neurons and glia could be distinguished by transmitters, receptors, or transporters. This provides an excellent starting point for more in‐depth analysis for a better understanding of the functional relevance of the snRNA‐seq findings, to identify functional glia subtypes and neurons, and to pinpoint important species differences and similarities for future studies. Finally, it will be very exciting to begin to evaluate ‘reactive glia’ and changes in neurons in response to gut stress, GI disorders, or diseases. Future studies can potentially integrate information from snRNA‐seq and validate findings with Patch‐seq analysis. Large databases of raw data available for further analysis from snRNA‐seq data in human enteric neurons or glia provide plenty of raw material to test novel hypotheses using retrospective analysis.

The study by Drokhlyansky et al. [11] provides a comprehensive single‐cell transcriptomic atlas of the human and mouse enteric nervous system (ENS), identifying various cell types and their gene expression profiles at an unprecedented resolution. The authors identified diverse populations of enteric neurons, glial cells, and other supporting cells in both humans and mice, highlighting the complexity and heterogeneity of the ENS. The study uncovered significant species‐specific differences in the ENS, including variations in the abundance and types of enteric neurons and glial cells between humans and mice. The authors also identified conserved gene expression patterns across species, suggesting shared fundamental mechanisms in ENS development and function. A prominent example is genes involved in neurotransmitter synthesis and signaling (i.e., ChAT and Tyrosine Hydroxylase, TUBB3—encoding beta‐III tubulin and MAP2—microtubule‐associated protein 2, GABBR2—gamma‐aminobutyric acid type B receptor subunit 2 and ADRB2—beta‐2 adrenergic receptor). Also, glial markers (i.e., s100B and GFAP) were shown to be expressed in both human and mouse ENS. Other genes conserved between species included genes essential for the development and maintenance of the ENS (i.e., RET, Sox10). Another study by May‐Zhang et al. [30] also identified shared and disparate subtypes across species. For example, differences in the expression of genes encoding neurotransmitters, receptors, and enzymes involved in neurotransmitter synthesis and degradation were observed. These differences may potentially contribute to species‐specific variations in gastrointestinal physiology and behavior.

Application of snRNA‐seq has revealed neuronal and glial diversity in the human ENS, uncovering species‐specific differences and similarities that are crucial for understanding human GI diseases. This high‐resolution analysis enables the identification of unique populations of glia and neurons, offering insights into human‐specific responses to gut stress and disorders, which may not be fully replicated in animal models and as such can be considered a proof‐of‐concept approach. Follow‐up studies in preclinical models of disease for targets shared in humans and animals may yield more definitive results that can be translated to humans with clinical relevance.

## 10 Developmental Studies of the Human ENS

The paper by Burns et al. [6] discusses the increasing focus over the past two decades on developing stem cell‐based therapies for enteric neuropathies. These are disorders and diseases affecting the enteric nervous system (ENS) of the gastrointestinal tract. The main idea behind stem cell therapy for enteric neuropathies is to transplant ENS progenitor/stem cells into the gut wall. The goal is to replace damaged or absent neurons and glia within the ENS, thereby restoring normal gut function. Obstacles that must be overcome to progress from successful preclinical studies in animal models to clinical applications of ENS stem cell therapies include issues related to safety, efficacy, scalability, and regulatory approval.

The study by McCann and Thapar [59] investigates the development of neuronal subtypes and the onset of evoked electrical activity in the human ENS. The ENS is crucial for gastrointestinal motility, but little is known about its ontogenesis in humans. Human fetal gut samples were characterized using various techniques, including immunohistochemistry, calcium imaging, RNA sequencing, and quantitative real‐time polymerase chain reaction (qRT‐PCR) analysis. Data generated provided insights into the development of neuronal diversity, electrical excitability, and network formation in the human ENS. Understanding these processes is essential for establishing functional enteric circuitry. Further studies using this model could enhance our understanding of congenital enteric neuropathies. Another study by Memic et al. [16] aimed to identify proteins that regulate the differentiation and network formation of the ENS during embryonic development. By comparing RNA expression profiles of the entire ENS, progenitor cells, and non‐ENS gut cells in mice. The researchers identified 147 transcription and signaling factors that varied in spatial and temporal expression during ENS development. Most of the identified factors in mice were found to be conserved in the human ENS, indicating potential relevance to human gastrointestinal function and disorders.

Human tissue studies of ENS development provide insights into neuronal diversity, electrical excitability, and network formation in ways that animal models may not fully replicate. Essentially, this is a proof‐of‐concept approach. These studies help identify key regulatory proteins and factors relevant to human gastrointestinal function and congenital enteric neuropathies, which are essential for advancing stem cell therapies for enteric disorders.

## 11 Application of Human Intestinal Biopsies to Study Pathogenesis of GI Disorders

Feasibility of nerve activity recordings in routine human intestinal biopsies was established by calcium neuroimaging in submucous neurons [18, 25, 31]. The translational value of the technique and its application in GI disorders was proven in functional dyspepsia (FD) [18]. Studies provided evidence for neuronal and structural changes in FD patients in submucous ganglia. Nasser et al. [25] discuss the use of human intestinal biopsies obtained during endoscopic procedures as an alternative approach to studying the pathogenesis of IBS. Unlike animal models, human biopsies allow for the examination of peripheral mechanisms directly in patients with IBS. Studies using human intestinal biopsies have provided insights into the biological activity of mediators within the mucosal tissue of IBS patients and alterations in the enteric neurons, extrinsic sensory pathways (dorsal root ganglia neurons), the immune system, and epithelial signaling in IBS patients compared to healthy subjects. Existing limitations should be overcome, such as sample size constraints and variability among patient populations.

Parkinson's disease (PD) is a neurodegenerative disorder characterized by both motor and non‐motor symptoms, including constipation. Live microscopy techniques were applied to routine duodenum biopsies to record neuronal calcium responses and mitochondrial membrane potential in nerve tissues. The study found no significant differences in enteric neuronal calcium responses, mitochondrial membrane potential, number, and volume between PD patients and controls [26]. Additionally, the numbers of neurons and ganglia in the biopsies were similar between the two groups. However, preserved neuronal functionality in PD patients suggests that GI symptoms may not arise from disturbed submucous neuronal function. The study suggests that GI symptoms in PD patients may be caused by impaired function of neurons in the myenteric plexus, which predominantly controls motility.

Using human intestinal biopsies offers distinct advantages over animal models by providing direct insights into neuronal activity and structural changes in patients. This approach enables the study of disease‐specific mechanisms, such as alterations in sensory pathways and immune responses, which may not be fully replicated in animals.

## 12 Live Imaging of Primary Neurons in Long‐Term Cryopreserved Human Nerve Tissues

The study by Fortea et al. [67] focuses on tissue cryopreservation as a solution to the scarcity of human nerve tissues, particularly live tissues. While brain tissue is typically only available postmortem, live nerve tissue, particularly from the intestine, can be obtained via endoscopic biopsy forceps. The study presents a method for preserving human primary neurons obtained from intestinal biopsies over longer periods of time. The use of a cryoprotective agent and controlled cooling are essential steps in properly storing the nerve tissue and enabling functional measurements after thawing. The preserved primary neurons were evaluated for functionality through calcium imaging that confirmed their viability. The study demonstrates that the human ENS serves as a realistic source of primary neurons that can be successfully preserved over long periods. This preserved tissue can be utilized for gastrointestinal‐specific as well as general neuroscience research, providing valuable insights into neural function and pathology.

The use of cryopreserved human nerve tissues may offer a significant advantage over animal models by providing a more relevant and sustainable source of human‐specific primary neurons for long‐term studies. Further validation will be needed to confirm the utility of the cryopreserved human nerve tissue in studies of normal or diseased gut.

## 13 Characterization of Projections of Longitudinal Muscle Motor Neurons in Human Colon

The ENS contains inhibitory and excitatory motor neurons that modulate intestinal smooth muscle contractility. The study by Humenick et al. [51] used retrograde tracing ex vivo with DiI, along with multiple labeling immunohistochemistry, to characterize motor neurons innervating the tenia and longitudinal muscle of the human colon. The most abundant immunohistochemical markers in the tertiary plexus were vesicular acetylcholine transporter, nitric oxide synthase (NOS), and vasoactive intestinal polypeptide (VIP). 95% of retrogradely traced motor neurons innervating longitudinal muscle were located within 6 mm oral or anal to the DiI application site. Excitatory motor neuron cell bodies, immunoreactive for choline acetyltransferase (ChAT), were clustered aborally, whereas NOS‐immunoreactive cell bodies were distributed on either side of the DiI application site. Cholinergic (ChAT)‐immunoreactive excitatory motor neurons to the tenia were clustered aborally, whereas NOS‐immunoreactive inhibitory motor neurons had both ascending and descending projections. VIP immunoreactivity was rarely present without NOS immunoreactivity in motor neurons.

Using human tissue provides a more accurate representation of human intestinal motor neuron projections for comparisons with animal models. DiI retrograde labeling is a powerful technique for further study of neuronal projections in different regions and nerve plexuses of the human gut.

## 14 Loss of Function Mutations and GI Symptoms

Loss of function mutations provide valuable information linking a specific channel, receptor, or protein to GI symptoms that can inform investigators about potential clinical significance. For example, loss of function of Piezo 2 channels [68] results in GI symptoms in patients that, in part, can be recapitulated by animal models with Piezo 2 deletion in DRG neurons.

Using human tissue allows for direct observation of the clinical relevance of loss of function mutations, such as those in Piezo 2 channels, and their link to GI symptoms. While animal models can recapitulate some aspects of these mutations, human tissues provide more accurate insights into how these mutations specifically impact human gastrointestinal function, leading to more clinically relevant findings and interpretation.

## 15 Concluding Remarks and Future Studies

**FIGURE 2:**
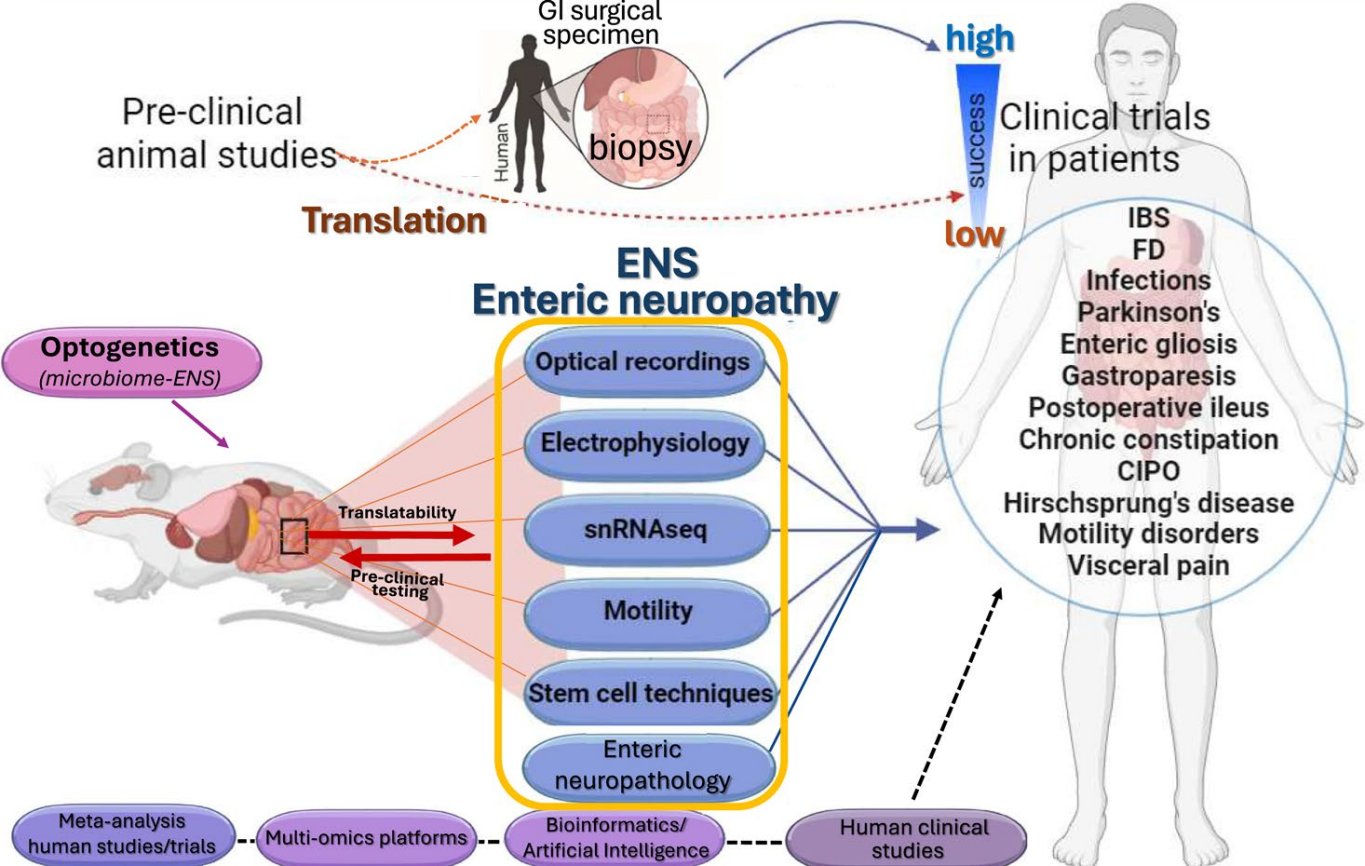
Translational pathways from animal models to human in vitro studies of the ENS and enteric neuropathies in gut disorders and diseases. The figure broadly illustrates the progression from pre‐clinical studies in animal models to human in vitro ENS models, using surgical specimens, biopsies, or isolated cells, as a key step in the complex pipeline leading to a new clinical trial on a novel therapeutic target. For example, human gut specimens are being used for the evaluation of enteric neuropathology, motility, optical recording from enteric neurons with voltage‐sensitive or calcium dyes, electrophysiological recording, snRNA‐seq analysis to dissect cell population‐specific gene signature profiles, stem cell regeneration, and bioengineering approaches or innovative use of optogenetics in preclinical models (i.e., human stem cells and microbiome‐mouse ENS studies). Such in vitro studies can be complemented by the incorporation of large clinical datasets from patients generated from clinical trial meta‐analysis, the application of multi‐omics platforms, and the use of bioinformatics/artificial intelligence tools. This integration of various research methodologies and clinical applications is one of the essential steps that might be considered in the eventual design of a clinical trial for disorders of gut‐brain interactions (DGBI) and GI diseases. After validating discovery studies done in animals in human samples (or, in some cases, completely replacing the animal studies with human sample‐based work), further supporting preclinical interventional studies in animal models may be needed.

Figure 2 is our overall scheme for an effective approach to developing a new drug from animal studies to clinical trials, depicting the various steps involved in the complex process. A critical step in the overall process is the direct animal‐to‐human link that requires validation of animal studies in human gut specimens and in vitro models, utilizing a number of novel in vitro translational models and techniques. Intuitively, everyone understands that a human is not always a good model for the mouse and vice versa. Notable species differences exist in the structure of the ENS, motility, physiology, microbiome composition, neuropathology, molecular signaling, transcriptome profiles, and pathophysiology. However, studies in humans are hampered by limited accessibility to human tissues for basic PhD scientists not in a clinical environment or department or not close to a hospital with access to human specimens. It may also be cost‐prohibitive to generate data in enough patients for vigorous statistical analysis and power of the study. Collaborations between basic scientists and clinician‐scientists are key to translational studies, and multidisciplinary teams of basic scientists, clinicians, surgeons, pathologists, and allied health professionals, and clinical research coordinators/assistants are instrumental in such studies. Having a PhD scientist in a clinical department and an experienced clinical research team provides a suitable environment for success in procuring viable human surgical specimens and biopsies for study. More support from extramural grant funding agencies is necessary for mechanistic translational studies on the human ENS, utilizing techniques developed over the past 25 years and support programs for the next generation of trainees in the field. Guidelines and a standardized approach to the use of human samples in translational studies are also needed. In conclusion, there is a critical need for further use of clinical samples to translate data in animals to humans in GI disorders and diseases before testing a potential therapeutic intervention in clinical trials. Routine availability of human gut specimens, in combination with technological advances and powerful new in vitro models of the human ENS from surgical tissue or biopsy specimens is providing new insights and advances in the field of neurogastroenterology and motility for GI disorders and diseases such as IBS, FD, postoperative ileus, chronic constipation, CIPO, infections, gastroparesis, Parkinson's Disease, Hirschsprung Disease, IBD, and visceral pain (see Figure 2). The current special issue of *Neurogastroenterology and Motility* highlights various aspects and mechanisms of enteric neuropathy.

## Author Contributions

Authors contributed to conceptualization, critical analysis, editing, final revision, designing and creating tables and figures and structuring the mini review. F.L.C. supervized the writing of this article. Authors take full responsibility for this mini review.

## Conflicts of Interest

The authors declare no conflicts of interest.

## Data Availability

Data sharing not applicable to this article as no datasets were generated or analysed during the current study.
